# Fueling fibrosis: BCAT1 links branched-chain amino acid metabolism to collagen production

**DOI:** 10.1172/JCI209543

**Published:** 2026-09-01

**Authors:** Marco Ronfini, John W. Elrod

**Affiliations:** Aging + Cardiovascular Discovery Center, Department of Cardiovascular Sciences, Lewis Katz School of Medicine at Temple University, Philadelphia, Pennsylvania, USA.

## Abstract

Fibrosis remains a major driver of organ dysfunction, yet the metabolic programs that sustain extracellular matrix production are incompletely understood. In this issue of the *JCI*, Takizawa and colleagues identified branched-chain amino acid transaminase 1 (BCAT1) as a crucial metabolic regulator of fibroblast activation and fibrosis in a model of cardiac fibrosis. Their observations were corroborated by analyses of datasets from patients with heart failure with preserved ejection fraction and metabolic dysfunction–associated steatohepatitis. They report that by coupling mechanical and TGF-β signaling to a proline biosynthesis and utilization program, BCAT1 enhanced collagen production in activated cardiac fibroblasts. These findings place branched-chain amino acid metabolism as a pivotal contributor to fibroblast activation and highlight BCAT1 as a promising therapeutic target for fibrotic disease.

## Collagen synthesis depends on proline availability

Fibrosis is characterized by excessive deposition of extracellular matrix (ECM), a pathological process that progressively disrupts tissue architecture and compromises organ function (1, 2). Whether it occurs in the heart, lung, liver, or kidney, fibrosis is driven by activated fibroblasts and myofibroblasts that acquire a remarkable capacity to synthesize and secrete ECM proteins, often referred to as a synthetic phenotype (1, 2). While decades of research has established central roles for TGF-β, inflammatory mediators, and mechanotransduction pathways in fibroblast activation (2), increasing attention has shifted toward a fundamental question: How do activated fibroblasts sustain the enormous metabolic demands required for continuous ECM production?

As fibroblasts differentiate into myofibroblasts, they undergo profound metabolic reprogramming that is marked by increased reliance on glycolysis and glutaminolysis to support the energetic and biosynthetic demands of matrix production (2–4). Approximately 20% of the amino acid composition of collagen consists of proline and hydroxyproline, making collagen synthesis highly dependent on mechanisms that ensure an adequate and sustained supply of this amino acid (5). In activated fibroblasts, proline can be generated de novo from glutamate through a pathway involving the sequential actions of the aldehyde dehydrogenase (ALDH18A1) and pyrroline-5-carboxylate reductase 1 (PYCR1). Glutamate itself can be derived from the branched-chain amino acids (BCAAs) valine (Val), leucine (Leu), and isoleucine (Ile) through transamination reactions catalyzed by BCAA transaminase 1 (BCAT1), a pyridoxal phosphate–dependent enzyme that transfers amino groups to α-ketoglutarate (5–7). In parallel, efficient collagen production also requires synthesis of prolyl-tRNA, a process mediated by glutamyl-prolyl-tRNA synthetase (EPRS), which has emerged as an important determinant of collagen translation in activated fibroblasts (8).

In this issue of the *JCI*, Takizawa and colleagues identified BCAT1 as a central regulator of this metabolic program (9). Their work positions BCAT1 at the intersection of BCAA metabolism and profibrotic signaling, revealing a mechanism through which fibroblasts acquire the metabolic capacity necessary to sustain collagen synthesis and tissue fibrosis. Interestingly, these findings are consistent with our group’s previous work identifying glutamine and glutamate as key metabolic determinants of fibroblast activation and differentiation, further supporting a central role for amino acid metabolism in fibrogenesis (3, 4, 10).

## BCAT1 marks the earliest stages of fibroblast activation

One of the most intriguing findings of the study by Takizawa et al. is the identification of BCAT1 as an early marker of fibroblast activation. They used an innovative suspension culture system to induce fibroblast dedifferentiation in myofibroblasts isolated from mouse hearts three days after experimental myocardial infarction (MI). They then induced reattachment-mediated differentiation and were thus able to compare gene expression in fibrotic, dedifferentiated, and redifferentiated myofibroblast states. The authors generated a transcriptional map of metabolic remodeling during myofibroblast differentiation to define fibroblast state transitions. Among 286 metabolism-related genes identified, only five were significantly upregulated during the early phase following MI, with *Bcat1* emerging as one of the most prominent candidates (9).

The induction of *Bcat1* was subsequently confirmed in multiple experimental settings featuring fibrosis. Indeed, *Bcat1* expression increased in response to both mechanical stimulation and TGF-β treatment in vitro and was elevated in cardiac fibroblasts isolated from MI hearts in vivo. Importantly, increased *BCAT1* expression was also observed in human heart failure with reduced ejection fraction (HFrEF) through analysis of the Gene Expression Omnibus (GEO) GSE161472 dataset. Intriguingly, Takizawa et al. observed a similar increase in metabolic dysfunction–associated steatohepatitis (MASH) by analyzing the GSE126848 dataset, suggesting that *BCAT1* induction represents a conserved feature of fibrotic remodeling across distinct organs and disease contexts.

Single-cell transcriptomics analyses provided further insight into the timing of *Bcat1* expression during fibroblast activation. In healthy hearts, *Bcat1* expression was largely absent from resident fibroblasts. Following MI, however, expression appeared in both α–smooth muscle actin–negative (α-SMA–negative) and α-SMA–positive fibroblast populations, indicating that *Bcat1* was induced before the acquisition of a fully differentiated myofibroblast phenotype. Consistent with this notion, *Bcat1* expression was detected in fibroblasts expressing the activation markers *Snail* and *Col1a1* at day 3 after MI, a stage preceding maximal SMA (*Acta2*) expression, which typically peaks later during scar formation. The positive correlation between BCAT1 and SNAIL expression in human HFrEF samples further supported the association.

These observations reinforce the emerging view that fibroblast activation is not a binary process but rather a continuum of transitional states. By identifying BCAT1 within the earliest activated fibroblast populations, Takizawa et al. confirmed that metabolic reprogramming was initiated before full myofibroblast differentiation and actively contributed to the acquisition of an ECM-producing phenotype (9).

## Linking amino acid metabolism to collagen production

BCAT1 has traditionally been studied in the context of BCAA metabolism, with established functions in cancer, immune regulation, cardiomyocyte and neuronal biology (11–15). Its role in fibroblasts, however, has remained largely unexplored. Takizawa et al.’s study now provides evidence that BCAT1 coordinates a broader metabolic network required for collagen synthesis.

Comparative analysis of *Bcat1*-positive and *Bcat1*-negative fibroblasts from the mouse MI model identified profound differences in genes associated with organonitrogen metabolism. Among the most downregulated genes in *Bcat1*-negative cells were *Aldh18a1*, *Pycr1*, and *Eprs*, three genes directly involved in proline production and utilization. Notably, these same genes were strongly induced during fibroblast activation in vitro and were elevated in human HFrEF and MASH samples.

Functional studies confirmed a causal relationship. Silencing *Bcat1* reduced expression of ALDH18A1, PYCR1, EPRS, and COL1A1 in MI-activated myofibroblasts, resulting in diminished intracellular proline levels and broad alterations in amino acid metabolism. Surprisingly, *Bcat1* knockdown did not affect α-SMA expression, indicating that BCAT1 selectively regulates the collagen-producing machinery rather than myofibroblast formation itself. In other words, fibroblasts can still acquire a myofibroblast-like phenotype in the absence of BCAT1, but they lose much of their capacity to sustain high-capacity collagen biosynthesis. Importantly, supplementation with exogenous proline partially restored collagen production following BCAT1 inhibition, supporting the concept that proline availability becomes rate limiting during matrix synthesis.

These findings reinforce the concept that fibroblasts do not merely activate fibrotic genes. Rather, they must simultaneously establish the metabolic infrastructure required to support the substantial biosynthetic demands of ECM production. BCAT1 emerges as a central coordinator of this metabolic adaptation, coupling amino acid metabolism to collagen synthesis.

## BCAT1 couples metabolism to profibrotic signaling

The study by Takizawa et al. becomes particularly interesting when addressing how a metabolic enzyme regulates the expression of genes involved in proline synthesis. The authors focused initially on SMAD3, a central downstream effector of TGF-β signaling and the quintessential regulator of fibrotic gene expression (9).

Loss of BCAT1 markedly reduced SMAD3 phosphorylation in MI-activated myofibroblasts, resulting in decreased expression of *Col1a1* and other profibrotic genes. Chromatin occupancy sequencing further demonstrated reduced SMAD3 binding to regulatory regions of *Aldh18a1*, *Pycr1*, and *Eprs* following *Bcat1* silencing, establishing a direct connection between BCAT1 activity and transcriptional control of the proline synthesis pathway.

Interestingly, BCAT1 overexpression alone was insufficient to activate SMAD3 or induce collagen expression in suspended fibroblasts, indicating that BCAT1 does not independently trigger fibrotic signaling. Rather, it appears to function as an amplifier of profibrotic stimuli, enabling fibroblasts to implement a collagen-producing program once activation has been initiated.

To identify the molecular link between BCAT1 and SMAD3, Takizawa et al. performed phosphoproteomics analyses and identified histone deacetylase 5 (HDAC5) as a critical intermediate. Specifically, phosphorylation of HDAC5 at Ser488 was markedly reduced following *Bcat1* knockdown. Consistent with this model, both *Hdac5* silencing and expression of a phospho-null HDAC5 mutant impaired SMAD3 phosphorylation and reduced collagen expression. However, the molecular mechanism by which HDAC5 promotes SMAD3 phosphorylation remains unclear, as it has not been fully established in the literature, nor was it directly addressed in the present study.

Importantly, supplementation with BCAAs restored both HDAC5 and SMAD3 phosphorylation in BCAT1-deficient fibroblasts, suggesting that BCAT1-dependent BCAA metabolism sustains a previously unrecognized HDAC5/SMAD3 signaling axis that drives expression of ALDH18A1, PYCR1, and EPRS.

While the molecular link between BCAA metabolism and HDAC5 phosphorylation remains unclear, this study provides a potential mechanism through which metabolic changes can regulate collagen production. An important next step will be to identify the kinase that phosphorylates HDAC5 and to understand whether BCAAs actively participate in signaling or primarily contribute as metabolic substrates.

## Targeting BCAT1 improves cardiac remodeling after MI

The translational implications of these findings are underscored by Takizawa et al.’s in vivo studies in mouse models of cardiac fibrosis (9). Genetic deletion of *Bcat1* reduced expression of *Aldh18a1*, *Pycr1*, *Eprs*, and *Col1a1* in murine hearts 3 days after MI and was accompanied by reduced phosphorylation of both HDAC5 and SMAD3. These molecular changes translated into meaningful functional benefits 28 days after MI: *Bcat1*-deficient mice exhibited reduced fibrosis and improved contractility compared with control animals.

Pharmacological inhibition of BCAT1 using the small molecule ERG240 produced similar results in mice. Treatment suppressed the expression of proline synthesis and utilization genes, attenuated SMAD3 activation, reduced collagen deposition, and preserved cardiac function. Importantly, therapeutic benefit was observed not only when treatment was initiated immediately after MI but also when administration began seven days following MI, by which point the acute inflammatory phase had largely resolved. This observation is particularly relevant from a clinical perspective. Most patients are treated after injury has already occurred, and interventions capable of targeting established fibrotic programs remain limited. The ability of BCAT1 inhibition to reduce fibrosis even when initiated after fibroblast activation has begun suggests that metabolic interventions may retain efficacy during later stages of disease progression.

## Metabolism takes center stage in fibrosis

Several questions remain. Because Takizawa et al.’s study relied primarily on global BCAT1 inhibition, lineage-specific approaches will be required to define the relative contribution of fibroblasts compared with other BCAT1-expressing cell populations. Moreover, fibrosis is not universally detrimental. Following myocardial infarction, scar formation is essential for maintaining structural integrity to avoid cardiac rupture (16), raising important questions regarding the therapeutic window within which BCAT1 inhibition can safely suppress pathological remodeling without impairing wound healing.

Finally, whether this mechanism extends to pulmonary fibrosis, chronic kidney disease, systemic sclerosis, or other fibrotic disorders remains to be determined. However, the observation that BCAT1 is elevated in both cardiac and hepatic fibrosis raises the intriguing possibility that BCAT1 represents a common metabolic dependency shared across diverse fibrotic diseases.

For decades, fibrosis research has focused primarily on cytokines, signaling pathways, and transcription factors. Here, Takizawa et al. add to a growing body of evidence demonstrating that metabolic reprogramming is not simply a consequence of fibroblast activation but an essential driver of fibrogenesis (2). Their work uncovers a connection between BCAA metabolism and collagen synthesis, highlighting how control of the metabolic supply chain may be just as important as regulation of the signaling pathways that initiate fibrosis.

## Conflict of interest

The authors have declared that no conflict of interest exists.

## Funding support

This work is the result of NIH funding, in whole or in part, and is subject to the NIH Public Access Policy. Through acceptance of this federal funding, the NIH has been given a right to make the work publicly available in PubMed Central.

- NIH grants R01HL172926, R01NS121379, and 2 P01HL134608-06A1 (to JWE).

## Figures and Tables

**Figure 1:**
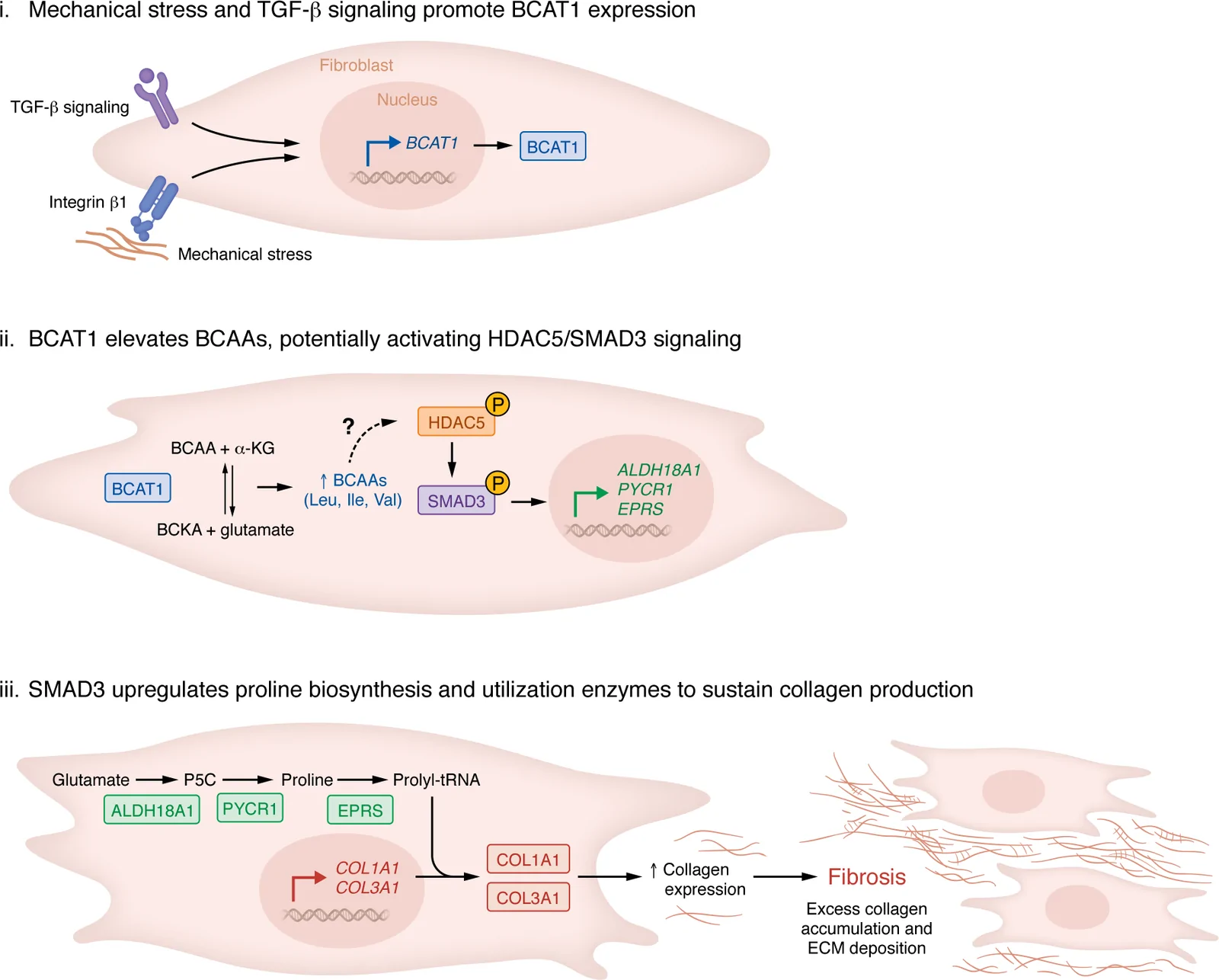
BCAT1 links BCAA metabolism to collagen production. Takizawa et al. (9) identified BCAT1 as a crucial metabolic regulator of fibroblast activation and fibrosis. Their findings support a mechanism by which (i) mechanical stress and TGF-β signaling induce BCAT1 expression in protomyofibroblasts. (ii) Subsequently, BCAT1-mediated BCAA metabolism potentially promotes phosphorylation of HDAC5 and activation of SMAD3, leading to increased transcription of ALDH18A1, PYCR1, and EPRS. (iii) These genes encode enzymes that enhance proline synthesis and utilization, supporting efficient production of collagens I and III and ECM deposition. Genetic or pharmacologic inhibition of BCAT1 disrupted this pathway, reducing fibrosis and improving cardiac function after MI.

## Version 1 09/01/2026

Electronic publication
